# Is restrictive fluid resuscitation beneficial not only for hemorrhagic shock but also for septic shock? A meta-analysis

**DOI:** 10.1097/MD.0000000000025143

**Published:** 2021-03-26

**Authors:** Shuaiyu Jiang, Mengmeng Wu, Xiaoguang Lu, Yilong Zhong, Xin Kang, Yi Song, Zhiwei Fan

**Affiliations:** a Graduate School; b Department of Emergency Medicine, Zhongshan Hospital, Dalian University, Dalian, China.

**Keywords:** hemorrhagic shock, limited fluid resuscitation, meta-analysis, septic shock

## Abstract

**Background:**

Whether to use limited fluid resuscitation (LFR) in patients with hemorrhagic shock or septic shock remains controversial. This research was aimed to assess the pros and cons of utilizing LFR in hemorrhagic shock or septic shock patients.

**Methods:**

PubMed, Cochrane Library, Embase, Web of science, CNKI, VIP, and Wan Fang database searches included for articles published before December 15, 2020. Randomized controlled trials of LFR or adequate fluid resuscitation in hemorrhagic shock or septic shock patients were selected.

**Result:**

This meta-analysis including 28 randomized controlled trials (RCTs) and registered 3288 patients. The 7 of 27 RCTs were the patients with septic shock. Others were traumatic hemorrhagic shock patients. Comparing LFR or adequate fluid resuscitation in hemorrhagic shock or septic shock patients, the summary odds ratio (OR) was 0.50 (95% confidence interval [CI] 0.42–0.60, *P* < .00001) for mortality, 0.46 (95% CI 0.31–0.70, *P* = .0002) for multiple organ dysfunction syndrome (MODS), 0.35 (95% CI 0.25–0.47) for acute respiratory distress syndrome (ARDS), and 0.33 (95% CI 0.20–0.56) for disseminated intravascular coagulation (DIC).

**Conclusion:**

Limited fluid resuscitation is the benefit of both traumatic hemorrhagic shock patients and septic shock patients.

## Abstract 2

Supplemental Digital Content is available in the text

## 1 Introduction

Shock is a syndrome that is caused by a strong pathogenic factor in the body, due to the sharp reduction of effective circulating blood volume, extensive, sustained, and significant reduction of blood flow perfusion, resulting in systemic microcirculatory dysfunction and serious disorders of vital organs.^[1]^ It accounts for about 30% to 40% of deaths in the first 24 hours after injury and is the leading cause of death in trauma patients.^[2]^ Hemorrhagic shock leads to a vicious circle, including hypothermia, acidosis, and coagulopathy—also known as lethal triad, which causes high mortality.^[3]^ Fluid resuscitation is especially important in shock resuscitation, but the administration of large amount of liquid contributes to and exacerbates the lethal triad and mortality. Hence, the concept of limited fluid resuscitation (LFR) was first put forward by Stern et al in 1992.^[4]^ Limited fluid resuscitation, also called permissive hypotension or hypotensive resuscitation, using of limited fluids and blood products during the early stages of treating hemorrhagic shock is a new resuscitation strategy. A lower-than-normal blood pressure is maintained until active bleeding is controlled.^[5]^ Many randomized controlled trials (RCTs) have also experimentally studied which is more beneficial for restrictive fluid resuscitation and adequate fluid resuscitation, but the results are not the same. The existing meta-analysis also faces problems such as insufficient sample size or low RCT quality. Conventional adequate fluid resuscitation faces various problems in hemorrhagic shock, so what about septic shock?

Sepsis is characterized by inflammation-induced endothelial dysfunction leading to vascular leakage and vasodilatation.^[6]^ In the new shock classification, septic shock belongs to distributed shock. It is not exactly the same as the mechanism of hemorrhagic shock, the cause is either a loss of regulation of vascular tone, with the volume being shifted within the vascular system, and/or disordered permeability of the vascular system with shifting of intravascular volume into the interstitium.^[7]^ Distributive shock, on the other hand, is a state of relative hypovolemia resulting from pathological redistribution of the absolute intravascular volume and is treated with a combination of vasoconstrictors and fluid replacement.^[8]^ Different therapeutic measures are needed for the different types of shock, so what does it take for a sufficient amount of fluid recovery?

In order to answer this question, we looked up the International Guidelines for Management of Sepsis and Septic Shock: 2016, it is recommended that the fluid resuscitation method for sepsis-induced hypoperfusion, administer at least 30 mL/kg of crystalloid per hour for the first 3 hours. However, despite their strong recommendations, the quality of evidence supporting these recommendations is low.^[9]^ Simultaneously, others hold that mortality in adult patients with septic shock increased at 12 hours and at 4 days as cumulative fluid balance increased and,^[10]^ similarly, increased daily fluid balances on the second day to seventh day have been associated with increased mortality in septic shock in adjusted analyses.^[11]^ This issue is controversial, however, few randomized controlled trials on hypotensive resuscitation existed in septic shock patients until recently.

The existing literature tells us the early fluid resuscitation can rely on increased venous return and cardiac output to enhance or maintain tissue perfusion. However, liquid administration may also give rise to deleterious effects by causing vital organs and tissue edema, resulting in organ dysfunction and impairment of oxygen delivery. Conversely, a restrictive fluid approach primarily limits the administration of fluid and relies on vasopressors to reverse hypotension and maintain perfusion.^[12]^ It is currently unknown whether a strategy using higher or lower fluid volume is better. There is a lack of strong data balance to creating clinical and scientific equipoise to confirm that one strategy is superior to another, notwithstanding there is some evidence to support the use of these two recovery strategies. In order to clarify this important issue, we examined the relationship between a range of shock patients and prognosis as mentioned in the relevant RCT, so we performed this meta-analysis.

## 2 Methods

This meta-analysis was conducted in the light of the preferred reporting items for systematic reviews and meta-analyses (PRISMA) guidelines.

### 2.1 Search methods

PubMed, Cochrane Library, Embase, Web of science, CNKI, VIP, and Wan Fang database searches included for articles published before December 15, 2020. In addition, the search for relevant primary literature and review the same topic to other retrospective studies. No language restrictions. The medical subject headings (MESHs) or keywords used in our search were as follows: “limited fluid resuscitation,” “restricted fluid resuscitation,” “hypotensive resuscitation,” “delayed resuscitation.” The terms above were used in combination with “shock” respectively. No language restrictions were imposed.

### 2.2 Inclusion and exclusion criteria

Studies included in this meta-analysis fulfilled the following criteria: population: patients in the study were diagnosed with shock; intervention: the intervention assessed was conventional fluid resuscitation with conventional liquid resuscitation (liberal fluid resuscitation) versus limited fluid resuscitation (delayed resuscitation or hypotensive resuscitation). And the method of liquid resuscitation is described definitely in these studies; design: available randomized comparative trials irrespective of publication status, language, or blinding.

Studies were excluded if they were: not RCT (case report, review, meta-analysis, or guideline); patients <18 years of age; patients were combined with traumatic brain injury (TBI) was excluded because of substantial clinical literature supporting the absolute prevention of hypotension in TBI patients; patients who were pregnant; not reporting detailed information for required clinical outcomes; study on animal observation (i.e., rat, pig, rabbit).

### 2.3 Types of outcome measures

The primary outcomes are: all-cause mortality, acute respiratory distress syndrome (ARDS), multiple organ dysfunctions (MODS), and disseminated intravascular coagulation (DIC).

Secondary outcomes included the rates of the following main postoperative morbidities: blood routine index (hemoglobin [Hb], platelet [PLT]), blood coagulation function (prothrombin time [PT], activated partial thromboplastin time [APTT]), blood gas analysis (base excess [BE], blood lactic acid [BLA]).

### 2.4 Assessment of risk of bias in included studies

The quality of the included RCTs was evaluated according to the methodological criteria of the Cochrane Handbook for Systematic Reviews of Interventions. We assessed the risks and bias in 7 areas, such as allocation sequence generation, allocation concealment, blinding of participants and study personnel, blinding of outcome assessors, management of incomplete outcome data, selective outcome reporting, and other potential sources of bias. When >10 studies were included in the results, the publication bias was assessed by the funnel plot. Grading of Recommendations Assessment, Development and Evaluation (GRADE) system was used to create a summary of findings table and assess evidence quality.

### 2.5 Statistical analyses

Statistical analysis is obtained using by RevMan Software (version 5.3, Cochrane Collaboration Network) and STATA software (version 12.0). Dichotomous variables were combined to estimate the pooled odds ratio (OR) with 95% confidence intervals (CIs). The *I*^2^ test was used to measure the statistical heterogeneity incorporated into the study, which we considered to be statistically significant heterogeneity when *P* < .1 or *I*^2^ > 50%. When no significant heterogeneity is observed, a fixed-effects model was used to make estimates, in other respects, we apply to use a random-effects model statistical analysis. *P* ≤ .05 indicated statistical significance in the integration results.

## 3 Result

### 3.1 Search results

**Figure 1:**
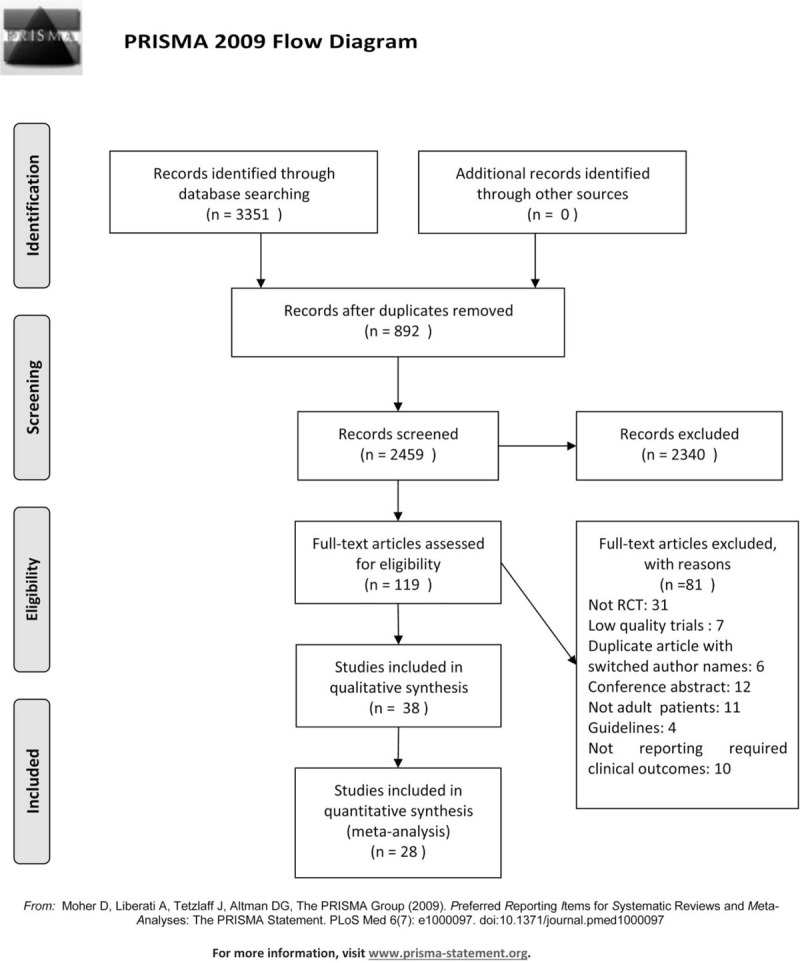
PRISMA diagram. PRISMA = preferred reporting items for systematic reviews and meta-analyses.

Figure PRISMA diagram illustrates a flow chart describing the article screening process, which was based on the PRISMA guidelines. We retrieved 3351 studies from PubMed, Cochrane Library, Embase, Web of science, CNKI, VIP, and Wan Fang database. After duplicates were identified and excluded, 892 were left. The case report, review, guideline, and meta-analysis according to the title or abstract were also excluded, leaving 2459 studies. Finally, including enrolled 3288 patients in 28 RCTs were included in this meta-analysis by intensive reading the full-text.^[5,13–39]^ (See Fig. 1).

### 3.2 Characteristics of trials included

We included 28 RCTs of published studies. The 7 of 28 RCTs were the patients with septic shock. Others were traumatic hemorrhagic shock patients (Table 1).

**Table 1:** Study characteristics.

Study	Country	Participants (LFR/RFR)	Intervention	Control	Infusion type
Bian Huimin 2013	China	Severe multiple hemorrhagic shock (50/48)	SBP maintaining above 80 mm Hg	SBP maintaining above 90 mm Hg	One intravenous channel for 6% hydroxyethyl starch sodium chloride injection (6 mL/kg),another for lactate ringer.
Bickell 1994	USA	Hypotensive patients with SBP ≤90 mm Hg and penetrating injures to the torso (289/309)	Delayed resuscitation with RLS 10 mL/h until definitive treatment	Immediate resuscitation to maintain SBP at least 100 mm Hg	Ringer's acetate solution
Carrick 2016	USA	Penetrating trauma patients with SBP < 90 mm Hg (84/80)	Keep MAP with 50 mm Hg	Keep normal MAP at least 65 mm Hg	Crystalloid, colloidal fluid, blood transfusion.
Chen Xiaoxiong 2008	China	Hemorrhagic traumatic shock with a survival time >72 h (25/27)	Limit fluid intake when infusion to SBP 70 mm Hg	Conventional resuscitation (SBP > 100 mm Hg)	Crystal and colloid ratio is 2–3:1
Corl 2019	China	Patients who were having severe sepsis or septic shock, per the Sepsis 2 International Consensus definitions (55/54)	Participants were permitted to receive up to 60 mL/kg of resuscitative IV fluids during the 72-h study period.	The usual care group received resuscitative IV fluid without any specified or suggested limits.	Resuscitative IV fluid included all IV crystalloid boluses (normal saline and ringers lactate) and maintenance IV fluid infusions (normal saline, ringers lactate, and sodium bicarbonate).
Dutton 2002	USA	Patients presenting in hemorrhagic shock (55/55)	Target SBP of 70 mm Hg	Target SBP > 100 mm Hg	Administration of crystalloid or blood products
Han Jiayu 2016	China	Hemorrhagic traumatic shock patients (34/34)	Maintain MAP 60–70 mm Hg	Maintain MAP 85–110 mm Hg	Crystal and colloid ratio is 2:1
Jiang 2019	China	Hemorrhagic traumatic shock patients with severe pelvic fracture (87/87)	Maintain MAP at 50–60 mm Hg and SBP above 70–90 mm Hg	Maintain MAP at 60–80 mm Hg and SBP above 100 mm Hg	The control group used plasma, suspended red blood cells, colloidal fluid and balance solution. The EFR group underwent hypertonic sodium chloride solution (7.5%)
Hjortrup 2016	USA	Patients with septic shock (75/76)	MAP below 50 mm Hg	Fluid boluses could be administered as long as the circulation	The choice of crystalloid solutions was at the discretion of the treating clinicians and maintained by the use of continuous infusion of norepinephrine.
Ling Jianzhong 2016	China	Traumatic hemorrhagic shock (20/20)	When SBP to 69 mm Hg and MAP reaches 50 mm Hg, slow down the infusion rate as appropriate.	Early rapid adequate fluid replacement, maintain 90 mm Hg for SBP.	Intermittent intravenous injection of cis-atracurium to maintain anesthesia.
Lu Bo 2015	China	Patients with acute upper gastrointestinal bleeding due to liver cirrhosis and concomitant hemorrhagic shock (27/24)	Maintain SBP ≥90 mm Hg, urine volume of 800 mL, and central venous pressure in a range of 5–12 cm H_2_O.	Maintain SBP 80–90 mm Hg, urine at 400–800 mL, and central venous pressure in a range of 5–12 cm H_2_O.	Ringer's solution and colloid hydroxyethl.
Li He 2015	China	Patients with septic shock (28/27)	Infusion of 500–1000 mL of fluid in a hour and maintain the MAP50–70 mm Hg. The amount of urine is maintained at about 0.5–1 mL/(kg h).	Infusion of 1000–1500 mL of fluid in a hour and maintain the MAP >70 mm Hg. The amount of urine is maintained at about 1–1.5 mL/(kg h).	Crystalloid and colloidal fluid.
Morrison 2011	USA	Patients in hemorrhagic shock (44/46)	Target mean MAP was 50 mm Hg	Manage with standard fluid resuscitation to a target MAP of 65 mm Hg.	Liquid, blood transfusion or vasopressor.
Macdonald 2018	Australian and New Zealand	Participants were adult patients presenting to the ED with suspected infection requiring IV antibiotic therapy who, in addition, had hypotension—defned as a systolic blood pressure (SBP) <100 mm Hg, (30/28)	Quickly infused to carry out fluid resuscitation and maintain the MAP at 50–70 mm Hg.	MAP was maintained at 70–90 mm Hg.	Infused by 2:1 balance solution and HS quickly.
Wang Afeng 2014	China	Thoracic and abdominal trauma combined with hemorrhagic shock (69/71)	MAP was kept at 55–65 mm Hg.	The rehydration was carried out quickly and sufficiently in the early before hemostasis, to keep the MAPat 80–90 mm Hg.	Hydroxyethyl starch and lactate ringer. Crystal and colloid ratio is 2–3:1.
Wang Bo 2016	China	Patients with severe thoracic trauma complicated with hemorrhagic traumatic shock (36/36)	When SBP rises to 70–75 mm Hg, slowing the infusion rate, and limit the amount of crystal liquid infusion to a certain extent.	Early, rapid and adequate infusion, SBP maintained above 90 mm Hg.	Plasma colloidal (706 generations) 500 mL and normal saline or Ringer solution 1000 mL. And the ratio of crystal glue is (2–3): l.
Wang Mei 2010	China	Patients with traumatic hemorrhagic shock (30/28)	Quickly infused to carry out fluid resuscitation and maintain the MAP at 50–70 mm Hg.	MAP was maintained at 70–90 mm Hg.	Infused by 2:1 balance solution and HS quickly.
Wang Qingxia 2016	China	Patients with septic shock (26/26)	Maintain MAP 50–60 mm Hg, urine volume is 0.5–1 mL/(kg h).	According to conventional liquid resuscitation therapy, maintain MAP ≥60 mm Hg, urine volume is 1–2 mL/(kg h)	Dopamine was used to maintain the lowest effective blood pressure. On the basis of this, liquid resuscitation is carried out by administering a crystal solution (physiological saline, lactated Ringer solution, etc) and a colloidal solution (albumin, hydroxyethyl starch, etc).Crystal and colloid ratio is 2:1.
Wen Zhenjie 2015	China	Patients with traumatic hemorrhagic shock (29/22)	Maintain SBP at around 75 mm Hg.	Early rapid fluid replacement until SBP exceeds 100 mm Hg	Colloid and electrolyte solution.
Xin Shaobin 2018	China	Patients with septic shock (48/40)	Reaching the target that CVP 8–12 mm Hg, SBP > 90 mm Hg, MAP ≥60 mm Hg, urine volume 1–1.5 mL/(kg h), ScvO_2_ > 70% or SVO_2_ ≥65% within 6 h.	Infusion of 1000–1500 mL of fluid in a hour during the first resuscitation	Resuscitation fluids include Ringer fluid, human serum albumin, crystalline fluid (0.9% sodium chloride solution, Ringer lactate solution, etc) and colloidal fluid (hydroxyethyl starch, etc). Crystal and colloid ratio is 1.5:1.
Xu Guoping 2015	China	Patients with uncontrolled hemorrhagic shock (60/60)	Put 7.5% NaCl was quickly administered in 4 mL/kg intravenously, and the amount of plasma was input. When the SBP was increased to 70 mm Hg, the MAP was maintained at 40–60 mm Hg, and the infusion rate was slowed down.	Follow in time and early 3: 1 ratio input balance solution and plasma. When the patient's SBP reaches 90 mm Hg, the mean arterial pressure is appropriately reduced at 60–80 mm Hg. Slowinfusion rate.	Control group ratio of input balance solution and plasma is 3:1. And the experimental group put 7.5% sodium chloride was quickly input in 4 mL/kg vein, and the amount of plasma was entered.
Xu Hang 2014	China	Sever traumatic sepsis and septic shock patients (30/30)	When the MAP rises to 50–60 mm Hg, reduced the fluid input and slow the infusion speed, maintained MAP at 50 mm Hg.	Maintain MAP 70 mm Hg	Antibiotic and vasoactive drug therapy.
Yan Lu 2018	China	Patients with multiple injuries in combination with shock (82/82)	The MAP was controlled between 40 and 50 mm Hg by controlling the infusion speed and volume of solution.	The MAP was kept between 60 and 80 mm Hg to ensure the blood supply of important organs such as heart and brain.	7.5% sodium chloride solution and plasma solution were infused.
Yao Jianhui 2015	China	Patients with uncontrolled hemorrhagic shock (43/43)	Maintain MAP 40–50 mm Hg	Maintain MAP 60–80 mm Hg	Lactate Ringer solution and hydroxyethyl starch (130/0.4) were input in a ratio of 3:1.
Zeng Fanyuan 2014	China	Patients with uncontrolled hemorrhagic shock (60/72)	Decreased infusion rate when the MAP rises to 50–60 mm Hg, maintain MAP around 50 mm Hg	Early, fast, adequate replenisher body, maintain MAP at 70 mm Hg	Apply sodium lactate, hydroxyethyl silicate powder 130/0.4 sodium chloride injection.
Zhao Shuangbiao 2007	China	Hemorrhagic traumatic shock (86/90)	According to blood pressure, faster than slower, so that blood pressure is maintained at 60–90/40–60 mm Hg.	Rapid rehydration to blood pressure exceeds 90/60 mm Hg	-
Zheng Weihua 2007	China	Patients hemorrhagic traumatic shock (60/72)	Decreased infusion rate when the MAP rises to 50–60 mm Hg, maintain MAP around 50 mm Hg	Maintain MAP 70 mm Hg	-
Zou Qiuping 2017	China	Hemorrhagic shock patients (49/49)	Controll the SBP at the level of 70–80 mm Hg	Maintenance of systolic pressure above 90–100 mm Hg as the standard	Hydroxyethyl starch as colloid solution and Ringer solution crystalloid liquid with a compound proportion of 1:2.

MAP = mean arterial pressure, SBP = systolic blood pressure.

### 3.3 Risk of bias in RCTs

**Figure 2:**
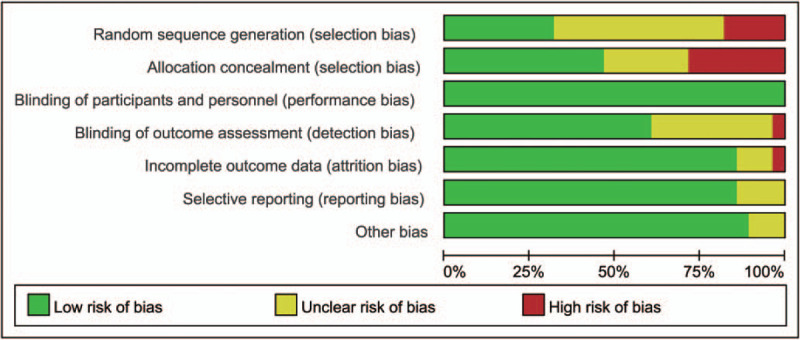
Risk of bias summary.

We evaluated the inclusion of RCTs based on 7 domains, such as allocation sequence generation, allocation concealment, blinding of participants and study personnel, blinding of outcome assessors, management of incomplete outcome data, selective outcome reporting, and other potential sources of bias to assess the risk of bias of blind method. Among 28 included RCTs, 13 studies supplied the detail allocation sequence generation method. (Fig. 2).

### 3.4 Synthesis of the primary outcome

#### 3.4.1 Mortality

**Figure 3:**
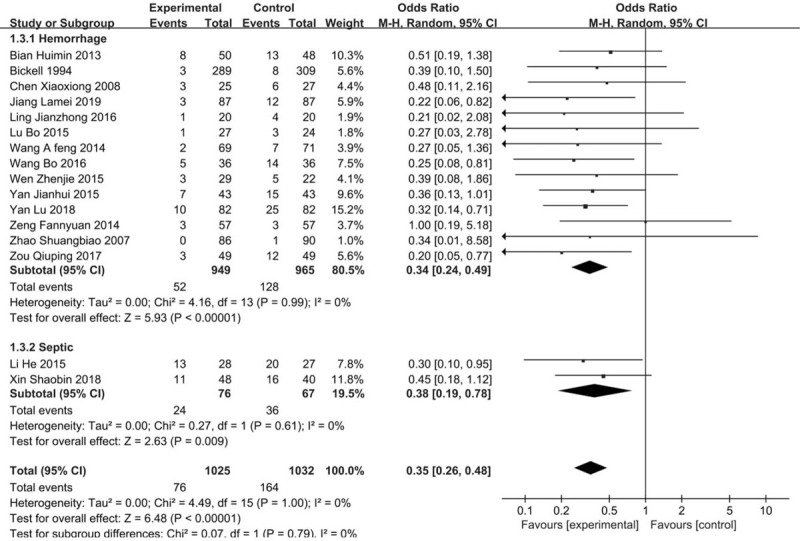
Forest plot of association between hypotensive resuscitation and normal resuscitation, relative to mortality.

Twenty-seven studies including 3233 patients compared the mortality of shock patients between LFR and regular fluid resuscitation (RFR). Among the 27 studies, 21 studies included 2674 patients with traumatic shock, showing that LFR mortality reduction is lower than RFR (OR 0.47, 95% CI 0.39–0.58), no significant heterogeneity was detected (*I*^2^ = 29%, *P* = .11), and had statistically significant (*P* < .00001); 6 included 559 patients with septic shock, showed an OR of 0.65 (95% CI 0.43–0.98). There was no significant subgroup difference between LFR or RFR subgroup (*I*^2^ = 42.4%, *P* = .19). (Fig. 3).

#### 3.4.2 Complication

##### 3.4.2.1 MODS

**Figure 4:**
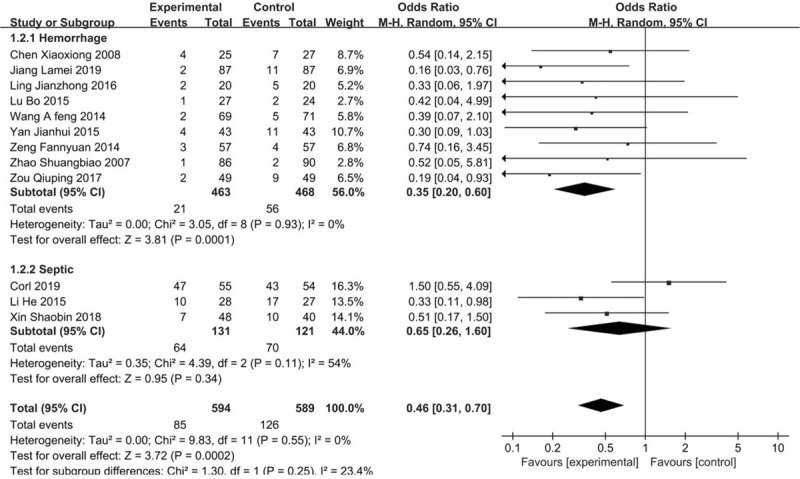
Forest plot of association between hypotensive resuscitation and normal resuscitation, relative to incidence rate of MODS. MODS = multiple organ dysfunctions.

Twelve studies with 1183 participants provided the incidence of MODS, and showed an OR of 0.46 (95% CI 0.31–0.70, *P* = .0002). We used a random effect model because significant heterogeneity was detected in involved studies. Subgroup analysis of 9 trials with LFR in traumatic patients showed a significant reduction of multiple organ dysfunction syndromes in the traumatic patients with RFR group, with an OR of 0.35 (95% CI 0.20–0.60, *P* = .0001). Simultaneously, there was a significant reduction in the 3 trials with septic shock patients (OR 0.65, 95% CI 0.26–1.60, *P* = .34). (Fig. 4).

##### 3.4.2.2 ARDS

**Figure 5:**
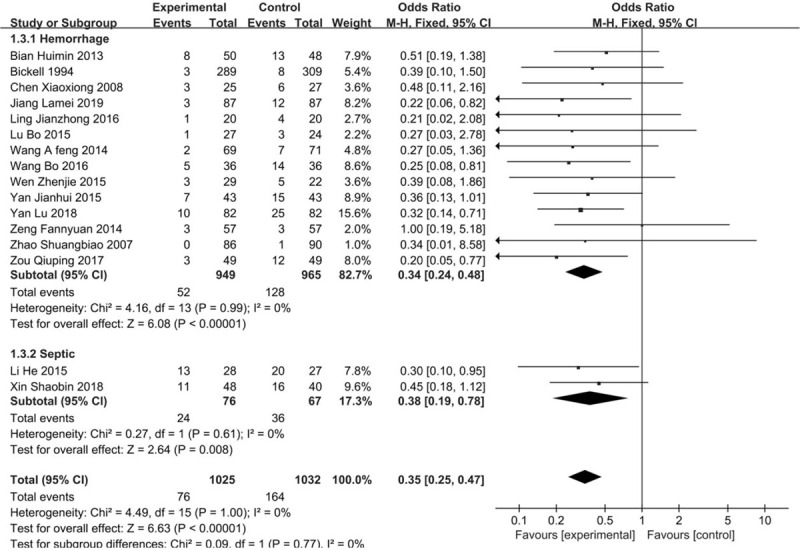
Forest plot of association between hypotensive resuscitation and normal resuscitation, relative to incidence rate of ARDS. ARDS = acute respiratory distress syndrome.

In total, 16 trials reported on the incidence of ARDS in the meta-analysis. In the stratified study, 14 articles were included in the hemorrhagic shock subgroups, and the OR was 0.34 (95% CI 0.24–0.48) with a significant reduction. Two trial with septic shock showed an OR of 0.38 (95% CI 0.19–0.78). There was no significant heterogeneity across all the studies (*I*^2^ = 0%, *P* = 1.00). (Fig. 5).

##### 3.4.2.3 DIC

**Figure 6:**
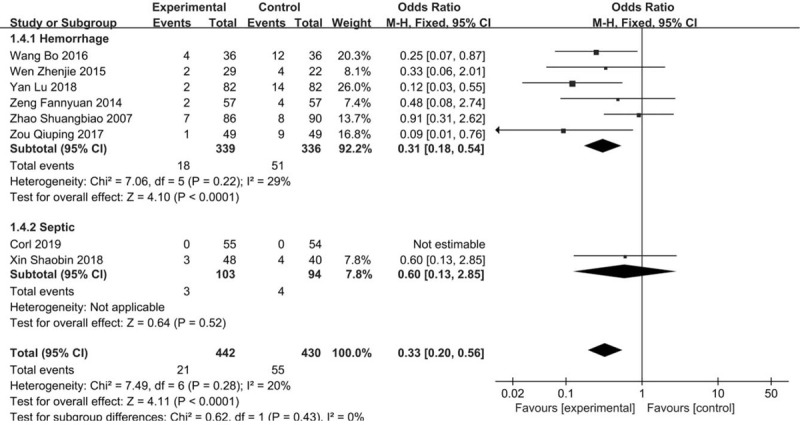
Forest plot of association between hypotensive resuscitation and normal resuscitation, relative to incidence rate of DIC. DIC = disseminated intravascular coagulation.

These adverse events were reported by 8 studies including 872 patients. The number of participants experiencing disseminated intravascular coagulation showed a reduction in the LFR group compared with RFR groups (OR 0.33, 95% CI 0.20–0.56). Six studies trials were stratified into hemorrhagic shock subgroups. A significant reduction was observed with an OR of 0.31 (95% CI 0.18–0.54). Two in septic shock subgroup was showed no significant reduction with OR of 0.60 (95% CI 0.13–2.85) and the difference was not statistically significant. No significant subgroup difference was found between hemorrhagic shock and subgroups septic shock (*I*^2^ = 0%, *P* = .43). (Fig. 6).

### 3.5 Synthesis of secondary outcome

#### 3.5.1 Blood routine index (Hb, PLT)

Nine trials investigated the hemoglobin (Hb) values after treated with LFR or RFR in hemorrhagic shock patients and showed a high heterogeneity across studies, which called for the random model to correct the bias. The overall effect showed that Hb value in LFR group was higher than that in the RFR group (MD = 12.09; 95% CI 0.73–23.45; *P* = .04). (Supplemental Digital Content Figure S1, http://links.lww.com/MD/F901) Seven trials including 1263 patients compared the PLT values between LFR and RFR groups. The heterogeneity test showed a severe degree of heterogeneity across studies, which need to select a random model for analysis. The overall effect suggested that platelet (PLT) value in LFR group was higher than that in the RFR group (MD = 19.65; 95% CI 2.44–36.85; *P* = .03). The Forest plot Funnel plot is provided in the Supplemental Content. (Supplemental Digital Content Figure S2, http://links.lww.com/MD/F902).

#### 3.5.2 Blood coagulation function (PT, APTT)

Twelve trials including 1719 patients compared prothrombin time (PT) while 5 trials including 435 patients compared activated partial thromboplastin time (APTT) between LFR and RFR groups. The heterogeneity test showed high heterogeneity in PT comparison but low heterogeneity in APTT (*X*^2^ = 3.56; *P* = .47; *I*^2^ = 0%). (Supplemental Digital Content Figure S3, http://links.lww.com/MD/F903) The overall effect suggested that RFR group prolong PT and APTT in hemorrhagic shock compared with that in LFR group (PT: MD = –4.32; 95% CI = –5.46 to –3.19; *P* < .00001 and APTT: MD = –4.98; 95% CI –5.79 to –4.18; *P* < .00001). The Forest plot Funnel plot is provided in the Supplemental Content. (Supplemental Digital Content Figure S4, http://links.lww.com/MD/F904).

#### 3.5.3 Blood gas analysis (BE, BLA)

Analysis of 6 trials (n = 640) investigating the base excess (BE) value after treated with LFR or RFR in hemorrhagic shock showed substantial heterogeneity across studies. With heavy heterogeneity, choose a random effects model to get stable results. And the BE value in the RFR group decreased more seriously than that in LFR group (MD = 1.65; 95% CI = –0.05–3.34; *P* = .06). (Supplemental Digital Content Figure S5, http://links.lww.com/MD/F905) Blood lactic acid (BLA), one of the traumatic lethal triad, were reported by 7 studies with 703 patients. Five of the 7 trials were stratified into hemorrhagic shock subgroups, and a significant reduction in blood lactic acid content (MD = –0.94; 95% CI –1.60–0.27; *P* = .006). The difference was statistically significant. Two trials with sepsis shock showed an OR of –0.60 (95% CI –1.08–0.11). There was no significant subgroup difference (*I*^2^ = 0%, *P* = 0.41). The Forest plot Funnel plot is provided in the Supplemental Content. (Supplemental Digital Content Figure S6, http://links.lww.com/MD/F906).

### 3.6 Publication bias

**Figure 7:**
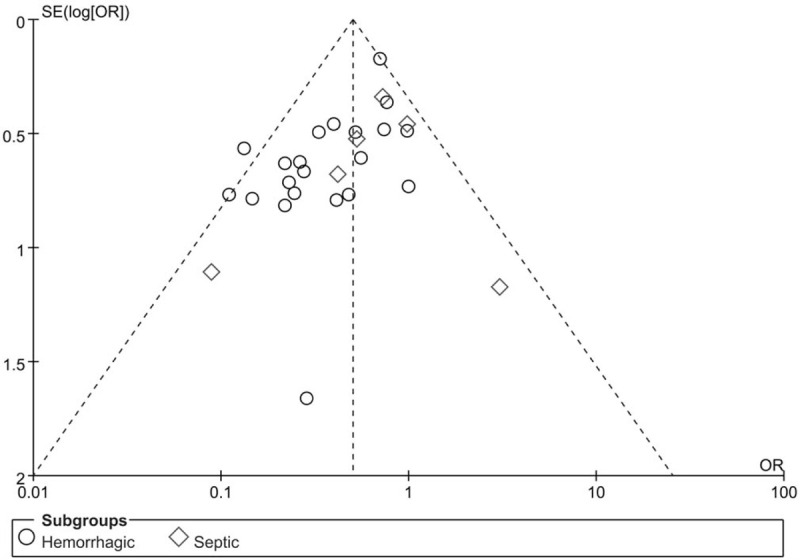
Funnel plot of association between hypotensive resuscitation and normal resuscitation, relative to mortality.

Funnel plots were drawn to test publication bias for mortality (Fig. 7). The result showed that the distribution of each research point was relatively symmetrical, which indicated that the possibility of publication bias was small. The same was true for publication bias in several other dichotomous outcome measures.

## 4 Discussions

This systematic review evaluated 28 RCTs and included 3288 patients with LFR or RFR. The RCTs examining RFR have demonstrated that RFR in the prehospital and hospital setting leads to more complications than hypotensive resuscitation, with divergent findings on the survival rates.^[35]^ The results demonstrate that LFR not only significantly reduces mortality, but may also be associated with decreased coagulopathy and other complications, including fewer MODS, ARDS, DIC, and shorter time of APTT and PT. The number of platelets is also seen to increase. And acidosis, one of the death triads, is also effectively controlled. Large doses of fluid resuscitation have been shown to cause tissue damage and microcirculation disorder, and even lead to ARDS and MODS, seriously affecting the prognosis of patients. Our results show that restrictive fluid resuscitation significantly reduces the incidence of these complications. The results of DIC, APTT, and PT showed that large doses of fluid resuscitation have been shown to be associated with blood thinning and clotting disease, causing the formation of blood clots to delay or destroy those that have already formed. Hb and PLT were closely related to intraoperative and postoperative blood transfusion volume, and the results showed that the intraoperative and postoperative blood transfusion volume was significantly reduced in patients undergoing restricted fluid resuscitation. Blood lactate levels were lower in the LFR group than in the RFR group, although there was no statistically significant difference in BE, which may indicate that limiting fluid resuscitation reduces the associated overdilution of blood, reduced oxygen supply to tissues and organs, and the risk of acidosis resulting from large doses of fluid resuscitation. This restrictive resuscitation policy is thought to minimize active bleeding while maintaining adequate organ perfusion and reducing the risk of coagulopathy. In patients with traumatic hemorrhagic shock, hypothermia and acidosis inhibit the generation of thrombin and the fibrinogen availability resulting in increased bleeding or prolonged bleeding time.^[40]^ Trauma patients are at the potential to lose body heat while at the scene of the injury already increased risk of hypothermia, through decreased heat production attributable to hemorrhagic shock and diminished oxygen consumption can improve survival.^[41,42]^ While in the case of septic shock patients, sepsis is often related to a deficiency in effective blood volume, leakage to the interstitial space, impaired function of blood flow into capillaries for exchange, and vasodilation. Hence, patients need to increase cardiac output and improve peripheral blood flow by large amounts of intravenous fluid.^[43]^ However, patients with sepsis though an increase in inflammation and endothelial dysfunction that decreased intake, increased additional losses because of higher vascular permeability. The subsequent distribution of fluid into the interstitium, in addition to third space losses, causes a lack of vascular responsiveness. When patients receive excess fluid during resuscitation efforts, they cause an increase in the capillary hydrostatic pressure and followed by a synergistic amount of fluid relocate into tissues. Organ dysfunction in various tissues of important organs such as the heart, kidneys and lungs, associated with this consequent edema.^[44]^ A positive fluid balance is harmful to organ function such as lung function and has been associated with increased time on prothrombin time. Furthermore, too much and too rapid fluid replacement will make the heart and lungs overburdened, which is not conducive to recovery. At last, restrictive fluid resuscitation allows the tissue to be in a low-pressure, low-perfusion condition for which can avoid ischemia-reperfusion damage.

There have been 2 meta-analyses about hemorrhagic shock in the past,^[45,46]^ and the meta-analysis is roughly the same as the previous 2 results. However, the scope of patients in this article has expanded, and the subgroup of patients with septic shock has been increased. Proposed a new perspective that restrictive fluid resuscitation is equally beneficial for septic shock. In addition, we conducted a more meaningful subgroup analysis and excluded many low-quality articles.

This meta-analysis had a certain amount of limitations. First, some included RCTs were not large the sample size and single center. The blinding was not addressed in all included RCTs, but we acknowledged that the blinding of different fluid resuscitation routes was impossible. Second, the resuscitation fluid selections were different. In our meta-analysis, the fluid of the RCTs was mainly saline, hydroxyethyl starch, lactated Ringer solution, etc. We have to acknowledge that some trials using colloidal resuscitation have fallen out of favor over the years because of poor results from large randomized controlled trials using resuscitation in critically ill patients. Meanwhile, in our meta-analysis, the patients have different degrees of shock varied greatly. Mild, moderate, severe shock patients all in. And several lesion severity scores appear in RCTs. Third, the limitations of this analysis include the fact that some clinical and methodological heterogeneities between the studies cannot be ruled out, and there may be some overtime bias. Last but not least, “Hypotensive resuscitation” is often referred to as an early restrictive fluid resuscitation strategy, but the timing of this “early” phase is not clearly defined. The time boundary of each trials is somewhat fuzzy (such as the time for hemostasis, the time for the onset of complications, etc), and some trials even do not mention the time at all. We hope there will be more articles in the future focusing on time nodes.

Since the populations studied in each randomized controlled trial are slightly different, as is the timing of intervention, targeted vitals, degree of shock, etc. There is still a need for a large, multicenter trial that can examine the benefit of hypotensive resuscitation in both trauma and septic shock patients.

## 5 Conclusions

The results of this meta-analysis revealed a significant benefit of hypotensive resuscitation both in traumatic hemorrhagic shock patients and septic shock patients. This benefit is not only reflected in mortality and complication rates, but also in reducing acidosis and coagulopathy, etc.

## Author contributions

**Conceptualization:** Shuaiyu Jiang, Xiaoguang Lu.

**Data curation:** Shuaiyu Jiang.

**Methodology:** Yi Song, Zhiwei Fan.

**Software:** Yilong Zhong.

**Supervision:** Xin Kang.

**Writing – original draft:** Shuaiyu Jiang.

**Writing – review & editing:** Mengmeng Wu.
